# Tissue bridges predict neuropathic pain emergence after spinal cord injury

**DOI:** 10.1136/jnnp-2020-323150

**Published:** 2020-08-11

**Authors:** Dario Pfyffer, Kevin Vallotton, Armin Curt, Patrick Freund

**Affiliations:** 1 Spinal Cord Injury Center, University Hospital Balgrist, Zurich, Switzerland; 2 Department of Brain Repair and Rehabilitation, UCL Institute of Neurology, University College London, London, United Kingdom; 3 Wellcome Trust Centre for Neuroimaging, UCL Institute of Neurology, University College London, London, United Kingdom; 4 Department of Neurophysics, Max Planck Institute for Human Cognitive and Brain Sciences, Leipzig, Germany

## Abstract

**Objective:**

To assess associations between preserved spinal cord tissue quantified by the width of ventral and dorsal tissue bridges and neuropathic pain development after spinal cord injury.

**Methods:**

This retrospective longitudinal study includes 44 patients (35 men; mean (SD) age, 50.05 (18.88) years) with subacute (ie, 1 month) spinal cord injury (25 patients with neuropathic pain, 19 pain-free patients) and neuroimaging data who had a follow-up clinical assessment at 12 months. Widths of tissue bridges were calculated from midsagittal T2-weighted images and compared across groups. Regression analyses were used to identify relationships between these neuroimaging measures and previously assessed pain intensity and pin-prick score.

**Results:**

Pin-prick score of the 25 patients with neuropathic pain increased from 1 to 12 months (Δmean=10.08, 95% CI 2.66 to 17.50, p=0.010), while it stayed similar in pain-free patients (Δmean=2.74, 95% CI −7.36 to 12.84, p=0.576). They also had larger ventral tissue bridges (Δmedian=0.80, 95% CI 0.20 to 1.71, p=0.008) at 1 month when compared with pain-free patients. Conditional inference tree analysis revealed that ventral tissue bridges’ width (≤2.1 or >2.1 mm) at 1 month is the strongest predictor for 12 months neuropathic pain intensity (1.90±2.26 and 3.83±1.19, p=0.042) and 12 months pin-prick score (63.84±28.26 and 92.67±19.43, p=0.025).

**Interpretation:**

Larger width of ventral tissue bridges—a proxy for spinothalamic tract function—at 1 month post-spinal cord injury is associated with the emergence and maintenance of neuropathic pain and increased pin-prick sensation. Spared ventral tissue bridges could serve as neuroimaging biomarkers of neuropathic pain and might be used for prediction and monitoring of pain outcomes and stratification of patients in interventional trials.

## Introduction

Spinal cord injury (SCI) is a devastating and life-changing event. In most cases, it produces immediate and permanent sensory, motor and autonomic dysfunction below the level of injury, resulting in a reduced quality of life.^1^ Along with the functional deficits, the majority of patients develops neuropathic pain (NP) at and/or below the level of injury.^2^ NP usually arises early after SCI,^2 3^ is often refractory to treatment^2 4 5^ and normally persists over years^3^ with a potentially increasing intensity.^6^ Although the origin of NP is largely unknown, it is thought to arise at least partially in the spinal cord.^7 8^ Preserved tissue bridges adjacent to the intramedullary lesion cavity, which can be identified in all patients with incomplete SCI,^9^ are permissive for electrophysiological information flow and the size of which is predictive of functional recovery.^9–11^ Importantly, NP below the level of injury develops over time.^12^ This suggests that fractions of sensory pathways within preserved tissue bridges become active over time and may affect both ascending and descending modulatory systems.^13–15^ Moreover, hyperactive dorsal horn neurons of the spinothalamic tract have been identified as a possible pathobiological substrate.^13^ Interestingly, recovery of spinothalamic tract function (eg, pin-prick score) is enhanced in patients with SCI suffering from NP and its magnitude has been associated with pain intensity.^16^

Based on neuroimaging, the location and width of spared midsagittal tissue bridges have been shown to be critically involved in the recovery of sensorimotor function.^9–11 17^ For example, dorsal tissue bridges covering the dorsal columns are predictive of sensory evoked potentials and recovery of light-touch function^11^ and may transmit pain signals evoked by normally non-painful stimuli (ie, allodynia).^18 19^ Ventral tissue bridges have been linked to recovery of motor function^11^ and cover, next to the anterior corticospinal tract,^20^ also portions of the anterior spinothalamic tract.^21^ Thus, the question arises whether width of ventral and dorsal tissue bridges can predict the emergence and/or maintenance of NP after SCI.

This study aimed to explore the value of spared midsagittal tissue bridges (ventral and dorsal) derived from conventional T2-weighted (T2w) serial scans^9–11^ in predicting the emergence and maintenance of SCI-related NP. Therefore, we investigated the relationships between the width of ventral and dorsal tissue bridges and pain intensity as well as its clinical characteristics.

## Methods

### Experimental design

**Figure 1:**
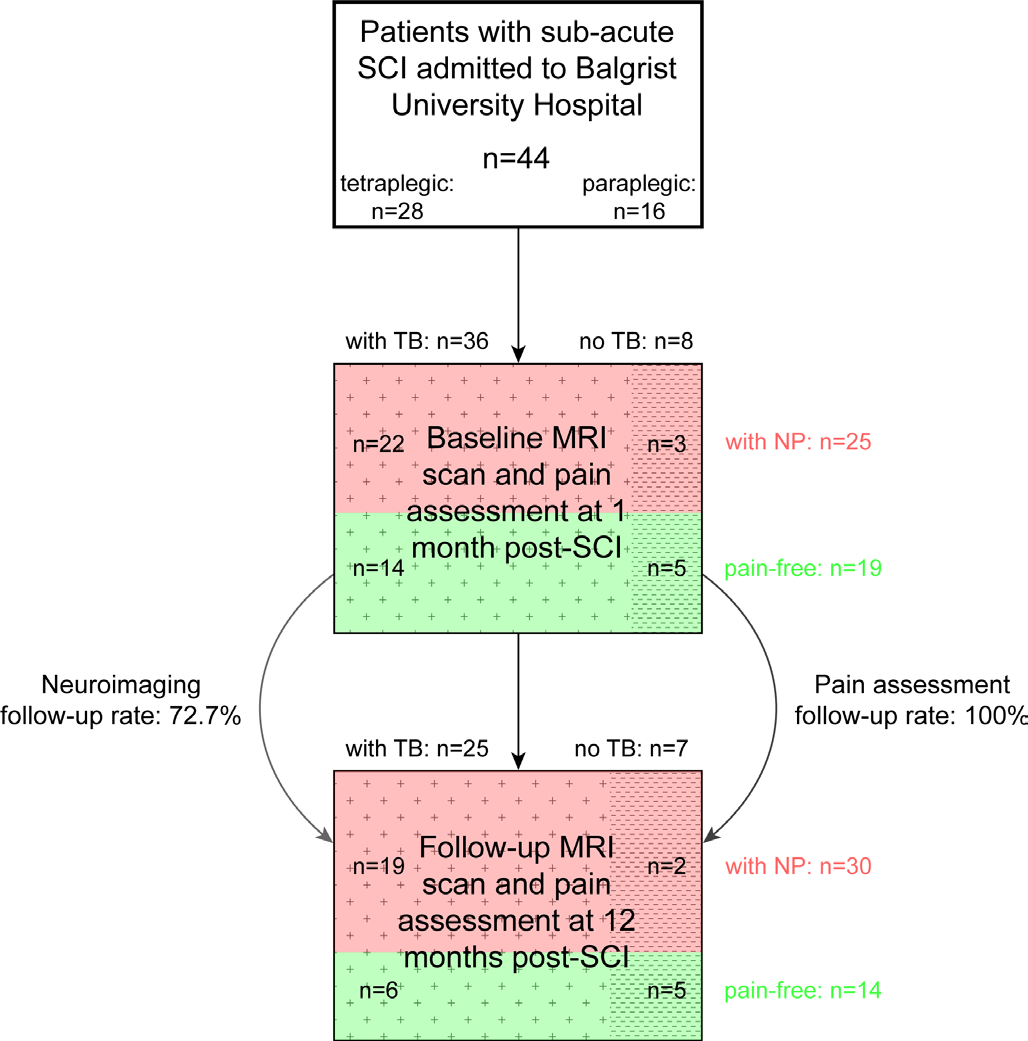
Study population with participant numbers, neuroimaging and pain characteristics as well as follow-up rate. Illustrative flow diagram showing the sample size of the study with subgroups according to their presence or absence of neuropathic pain (NP) and tissue bridges (TB). From 44 patients with subacute spinal cord injury (SCI), all had a follow-up pain assessment at 12 months, whereas only 32 underwent a follow-up MRI scan.

In this retrospective study, we included 44 patients with subacute SCI (28 tetraplegic and 16 paraplegic patients) who were admitted consecutively to the Balgrist University Hospital (Zurich, Switzerland) between May 1996 and January 2019. Of these 44 patients, all had a follow-up clinical assessment and 32 had a neuroimaging follow-up visit at 12 months post-SCI (figure 1).

We used 1 month and 12 months neuroimaging, neuropathic pain and clinical data from these patients to investigate group differences and clinicopathological relationships as well as outcome predictions by means of regression analysis and unbiased recursive partitioning (URP). Variables of interest included were lesion parameters as well as pain and clinical outcome measures at 1 month and 12 months follow-up.

We excluded patients with pre-existing neurological or mental disorders or brain lesions, as well as patients with MRI contraindications.

Tissue bridge data from a small subset of the study population was previously reported in a different context.^9–11^

### Clinical assessments

All patients were clinically examined using a comprehensive clinical protocol including 1) the International Standards for Neurological Classification of Spinal Cord Injury protocol^22^ and 2) the European Multicenter Study about Spinal Cord Injury (EMSCI) pain questionnaire (V.4.2, http://www.emsci.org/). On the EMSCI questionnaire, patients rate various aspects of pain (eg, current pain intensity, mean and maximal pain intensity during the last week before the assessment, location and quality of pain, intensity of allodynia and paraesthesia). The pain intensity was quantified using an 11-point numeric rating scale with ‘0’ indicating no pain and ‘10’ indicating the worst imaginable pain. Data from the EMSCI pain questionnaire was available for 43 patients with SCI at 1 month (24 patients with NP, 19 pain-free patients) and 42 patients at 12 months post-SCI (28 patients with NP, 14 pain-free patients). One patient and two patients without EMSCI pain questionnaire at 1 month and 12 months, respectively, reported to suffer from NP.

### Image acquisition

All study participants underwent MRI on 1.5 or 3 T Philips (Philips Healthcare, Best, The Netherlands), Siemens (Siemens Healthcare, Erlangen, Germany) or GE (GE Medical Systems, Waukesha, Wisconsin, USA) scanners. A 32-channel receive spine coil integrated in the table was used with all scanners. The anatomical MRI protocol consisted of standard sagittal T1-weighted, sagittal T2w and axial T2w clinical scans obtained at the lesion level. Repetition time (TR), echo time (TE) and flip angle (FA) of the sagittal T2w clinical sequences were used as follows: at 1.5 T (TR=4138 ms; TE=109 ms; FA=149 degrees) and at 3 T (TR=3938 ms; TE=97 ms; FA=153 degrees). The echo train length was 19 at 1.5 T and 17 at 3 T. At 1.5 and 3 T, the field of view9 was set to 330 mm×330 mm and 220 mm×220 mm, respectively. To reduce metal artefacts, readout bandwidths were increased at 1.5 T (415 Hz/pixel) and at 3 T (751 Hz/pixel). The spatial resolution of sagittal T2w images was 0.55 mm×0.55 mm×2.75 mm at 1.5 T and 0.57 mm×0.57 mm×2.75 mm at 3 T. The midsagittal slices of sagittal T2w scans were the ones used for lesion segmentation analysis.^9–11^

### Image analysis

Together with oedema and haemorrhage, intramedullary damage manifests as changes of signal intensity on T2w scans.^23^ Crucially, hyperintense signal changes in the subacute phase reliably reflect neural damage within the spinal cord^9 10^ rather than oedema and haemorrhage which is harder to differentiate in the acute phase after SCI. Patients’ neuroimaging data were only included if T2w scans showed a clearly visible lesion (ie, hyperintense signal)—if present—on the midsagittal slice whereas scans with insufficient image quality or lesion visibility due to metal artefacts were excluded.^9–11^ Neuroimaging data with appropriate image quality and lesion visibility was available for 44 patients with subacute SCI of which 32 had a follow-up scan at 12 months post-SCI.

Lesion segmentation was performed manually and blinded to patient identity and scan time point by rater DP. We used the Jim software (V.7.0, Xinapse Systems, Aldwincle, UK) to delineate the lesion on the midsagittal slice from sagittal T2w scans. This enabled us to assess the lesion area, its rostro-caudal lesion length, anterior-posterior lesion width and ventral and dorsal tissue bridges, the sum of both reflecting the total width of tissue bridges (ie, hypointense regions between the relatively hyperintense adjacent cystic cavity within the spinal cord and the cerebrospinal fluid).^9 10^

### Statistical analysis

Statistical analysis was performed using Stata software (V.4.2; StataCorp, College Station, Texas, USA). We used unpaired two-tailed t-tests to compare patients with SCI with NP and pain-free patients with SCI regarding their age at 1 month scanning time point and the time interval between injury and 1 month scan. Paired two-tailed t-tests were used for comparison of midsagittal tissue bridges (ie, total, ventral and dorsal), pin-prick score and mean pain intensity at 1 month and 12 months post-SCI.

We applied Mann-Whitney U tests for pairwise comparisons of patient groups regarding their pin-prick score and width of midsagittal tissue bridges (ie, ventral, dorsal and total) at 1 month and 12 months post-SCI as well as rate of change in pin-prick score from 1 month to 12 months.

We used multivariable linear regression analysis to investigate associations between imaging measures (ie, width of ventral midsagittal tissue bridges) and mean pain intensity at 1 month. One month quantitative lesion measures were also used with the same model to predict mean NP intensity and recovery of pain sensation (ie, pin-prick score) at 12 months follow-up. Age and sex were included as covariates of no interest in the regression models to adjust for linear age and sex dependency. Models including ventral tissue bridges were also corrected for dorsal tissue bridges. Prediction models were furthermore adjusted for 1 month clinical scores (eg, pin-prick score at 1 month). Potential confounders were only retained if the covariates were significant or if they had a substantial effect on the correlation coefficient of interest. Results were regarded as significant when the p values were ≤0.05 and CIs were set to 95%.

We further used a widely used^24–28^ unbiased recursive partitioning technique called conditional inference tree (URP-CTREE)^29^ implemented in the ‘party’ package within R^30^ (V.3.4.3). URP-CTREE performs a prospective prediction-based stratification of a patient population with regard to predefined outcome variables. URP-CTREE is a tree-structured regression model for independence tests between sets of predictors (eg, early imaging parameters) and specified future clinical end points (eg, pain sensation measures). We used 1 month ventral and dorsal midsagittal tissue bridges’ widths as predictors for mean neuropahtic pain intensity and pin-prick score at 1 month and 12 months. The URP-CTREE algorithm dichotomously separates an initial heterogeneous patient population into more homogeneous and well-defined pairs of subgroups (ie, nodes) with respect to the future clinical outcomes specified. Subgroups that will be further separated are referred to as inner nodes. Separation goes on as long as one of the predictor variables is found to be associated with the predefined clinical end point and to split the group into two disjoint subgroups with a p value ≤0.05. The algorithm is designed in a way that every separation is based on the singular most significant predictor with the aim to maximise the difference between the newly formed subgroups. Splitting groups is continued as long as there is any significant predictor value.

### Data availability

Anonymised data of this study are available on request from the corresponding author.

## Results

### Demographics and clinical characteristics

Forty-four patients with SCI (n=35 men (79.5%)) with a mean (±SD) age of 50.05±18.88 years were included. Patients with NP (50.40±17.38 years, n=25) and pain-free patients (49.58±21.17 years, n=19) did not differ in their age at 1 month (Δmean=−0.82, 95% CI −12.55 to 10.91, p=0.888). The patients’ time interval between injury and 1 month scan (ie, subacute stage) was 32.95±16.85 days. There was no difference in time since injury (Δmean=4.80, 95% CI −5.56 to 15.17, p=0.355) between patients with NP (30.88±17.55 days, n=25) and pain-free patients (35.68±15.93 days, n=19).

Over time, NP developed in six initially pain-free patients whereas one patient with NP at baseline reported to be NP-free at follow-up. Patients with NP at 1 month (56.8%) and 12 months follow-up (68.2%) reported a similar mean NP intensity of 3.67±1.61 and 3.68±1.61 (Δmean=0.01, 95% CI −0.89 to 0.91, p=0.979), respectively. Pin-prick score of patients with SCI with NP was 70.08±28.40 at 1 month and increased to 80.16±27.88 at 12 months post-SCI (Δmean=10.08, 95% CI 2.66 to 17.50, p=0.010, n=25). In pain-free patients, 1 month pin-prick score was 57.84±25.02 and did not significantly change over time (60.58±27.25 at follow-up (Δmean=2.74, 95% CI −7.36 to 12.84, p=0.576, n=19)). Between both patient groups, there was no difference in pin-prick score at 1 month (Δmedian=15.00, 95% CI −7.00 to 29.00, p=0.184) and the rate of change in pin-prick score from 1 month to follow-up at 12 months did not differ (Δmedian=3.00, 95% CI −3.00 to 12.00, p=0.286).

### The role of midsagittal tissue bridges in NP

Of 44 patients, 8 (ie, American Spinal Injury Association Impairment Scale (AIS) A patients) had no midsagittal tissue bridges (18.2%) while the remaining 36 (ie, 1 AIS A and 35 AIS B–D patients) did have midsagittal tissue bridges (81.8%) with an average width of 2.21±1.65 mm. Thirty-one of 44 (70.5%) patients had ventral tissue bridges with an average width of 1.17±1.18 mm and 29 of 44 (65.9%) had dorsal tissue bridges with an average width of 1.03±1.20 mm. Twenty-four patients had both ventral and dorsal tissue bridges while seven and five patients had only ventral or dorsal bridges, respectively.

**Figure 2:**
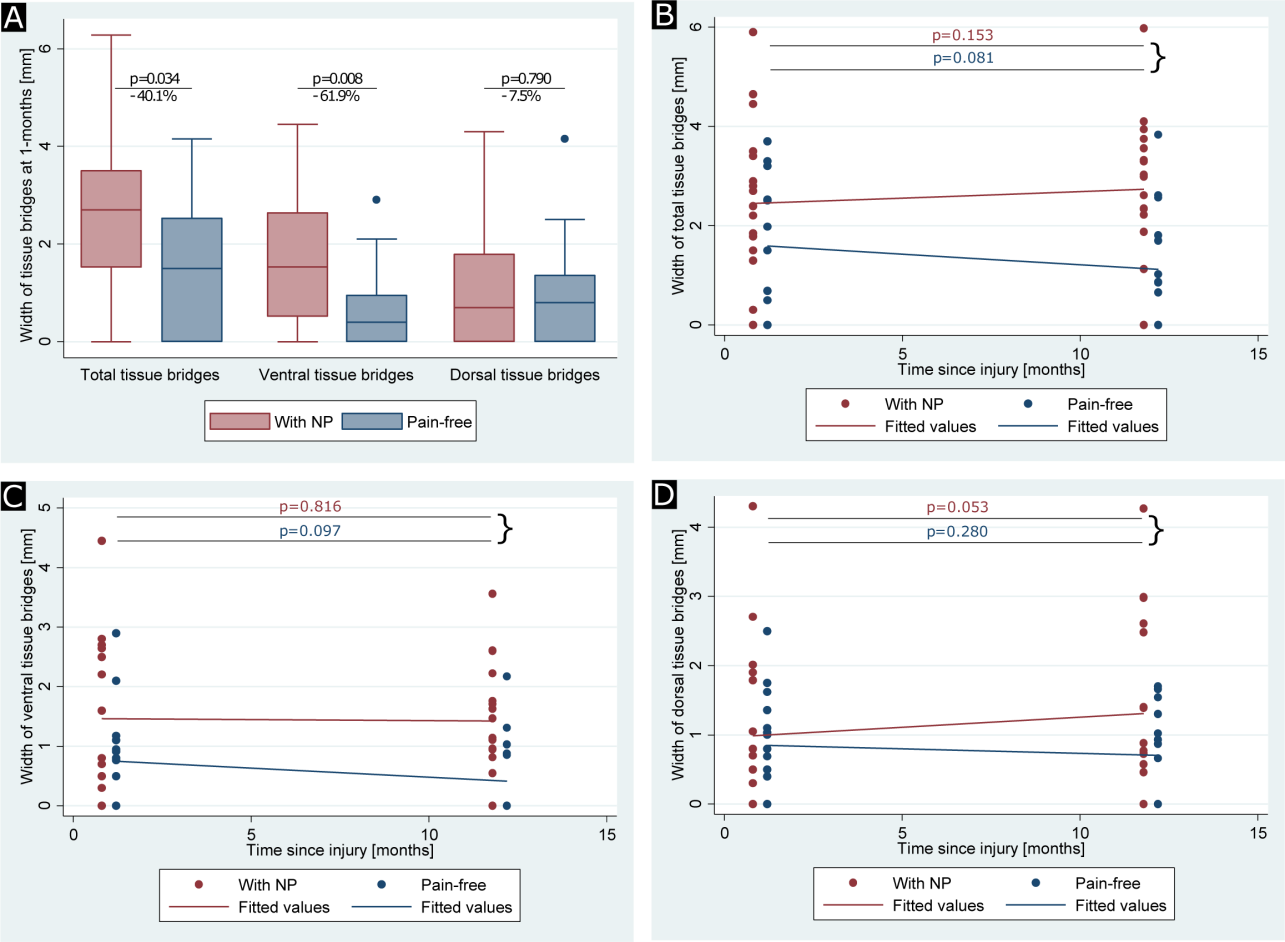
Between and within group comparison of tissue bridges at 1 month and 12 months. (A) Group differences of total, ventral and dorsal tissue bridges are shown for patients with spinal cord injury with neuropathic pain (NP) (indicated in red) and pain-free patients (indicated in blue). (B–D) Spatiotemporal evolutions of (B) ventral, (C) dorsal and (D) total tissue bridges from 1 month to 12 months are shown for patients with spinal cord injury with NP (indicated in red) and pain-free patients (indicated in blue) separately. Uncorrected p values are reported for significant differences.

At 1 month, width of total midsagittal tissue bridges was larger (Δmedian=1.15, 95% CI 0.00 to 2.12, p=0.034, figure 2A) in patients with SCI with NP (2.67±1.71 mm, n=25) when compared with pain-free patients (1.60±1.38 mm, n=19). At 1 month, ventral tissue bridges of patients with NP (1.60±1.25 mm, n=25) were larger (Δmedian=0.80, 95% CI 0.20 to 1.71, p=0.008) in comparison to pain-free patients (0.61±0.81 mm, n=19). Dorsal tissue bridges at 1 month were similar (Δmedian=0.00, 95% CI −0.69 to 0.50, p=0.790) in patients with NP (1.07±1.33 mm, n=25) and pain-free patients (0.99±1.03 mm, n=19).

Of 25 patients with SCI with NP and 19 pain-free patients, 17 and 15 had imaging follow-up data, respectively (table 1). In the 17 patients with NP, width of total tissue bridges was similar at 1 month and 12 months post-SCI (Δmean=0.29 mm, 95% CI −0.12 to 0.69 mm, p=0.153, figure 2B). Within the pain-free group, width of total tissue bridges was also comparable between 1 month and 12 months follow-up (Δmean=−0.48 mm, 95% CI −1.02 to 0.07 mm, p=0.081). Width of ventral tissue bridges was comparable at 1 month and 12 months in patients with NP (Δmean=−0.04 mm, 95% CI −0.39 to 0.31 mm, p=0.816, figure 2C) and pain-free patients (Δmean=−0.33 mm, 95% CI −0.73 to 0.07 mm, p=0.097). Similarly, the rate of change in width of dorsal tissue bridges over time was similar for patients with NP (Δmean=0.32 mm, 95% CI 0.00 to 0.65 mm, p=0.053, figure 2D) and pain-free patients (Δmean=−0.15 mm, 95% CI −0.43 to 0.13 mm, p=0.280).

**Table 1:** Dynamic change of width of total, ventral and dorsal tissue bridges

	Patients with SCI with NP (n=17)		Pain-free patients with SCI (n=15)	
Width of tissue bridges	1 month	12 months	1 month	12 months
Total, mean (SD), mm	2.45 (1.64)	2.73 (1.48)	1.60 (1.33)	1.12 (1.17)
Ventral, mean (SD), mm	1.46 (1.29)	1.42 (0.94)	0.75 (0.86)	0.42 (0.68)
Dorsal, mean (SD), mm	0.99 (1.20)	1.31 (1.29)	0.85 (0.74)	0.70 (0.66)

NP, neuropathic pain; SCI, spinal cord injury.

### Association of tissue bridges and clinical pain measures at 1 month

**Figure 3:**
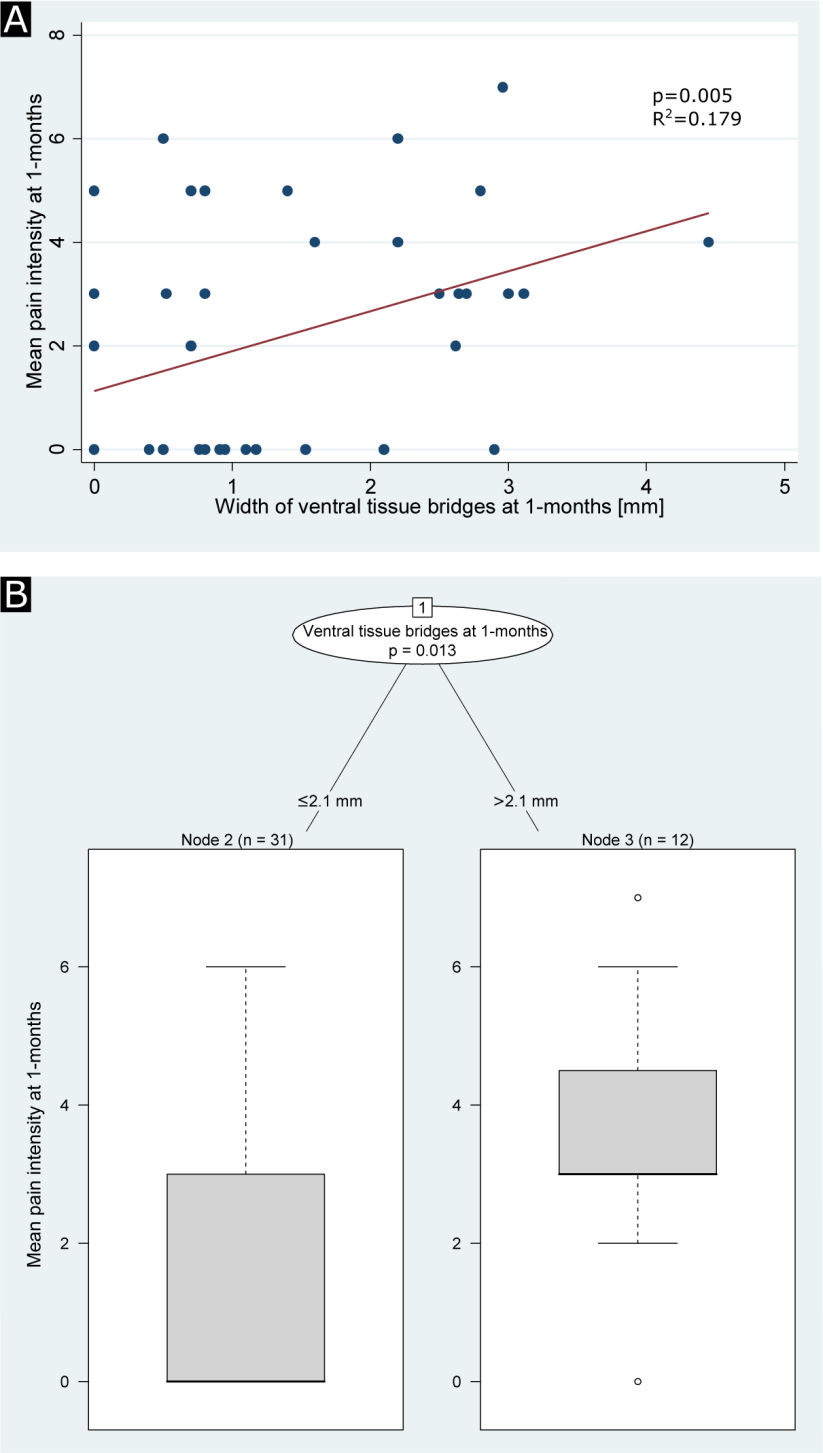
Regression analyses between width of tissue bridges and mean pain intensity at 1 month. (A) Regression graph showing the association of mean pain intensity at 1 month with width of ventral tissue bridges at 1 month for all patients with spinal cord injury. The red line indicates the linear fit. (B) Unbiased recursive partitioning conditional inference tree for the clinical end point mean pain intensity at 1 month of patients with spinal cord injury with available pain intensity data at 1 month (n=43). Ventral and dorsal tissue bridges’ widths at 1 month were used as predictors in the model. The algorithm led to a partition of the initial patient population (node 1) into two terminal nodes (nodes 2 and 3) representing the more homogeneous subgroups. Box plots at the bottom show sizes of the subgroups, indicated above each terminal node, with their corresponding distributions (including two-sided error bars) of the clinical end point. Uncorrected p values are reported in (A) and multiple testing-corrected (ie, Bonferroni-corrected) p values are reported in (B) for significant differences.

Larger width of ventral tissue bridges at 1 month was associated with a higher mean pain intensity at 1 month (coefficient=0.23, 95% CI 0.07 to 0.39, p=0.005, R^2^=0.179, n=43, figure 3A), adjusted for dorsal tissue bridges and age.

URP-CTREE analysis led to a partition of the entire cohort (p=0.013, n=43, figure 3B) into two terminal nodes with regard to the patients’ mean pain intensities (ranging from 0 to 10) at 1 month, according to the 1 month width of ventral tissue bridges being ≤2.1 mm (n=31, node 2) or >2.1 mm (n=12, node 3). The two subgroups presented 1 month mean NP intensities of 1.45±2.05 and 3.58±1.83, respectively.

### Association of tissue bridges and clinical pain measures at 12 months

**Figure 4:**
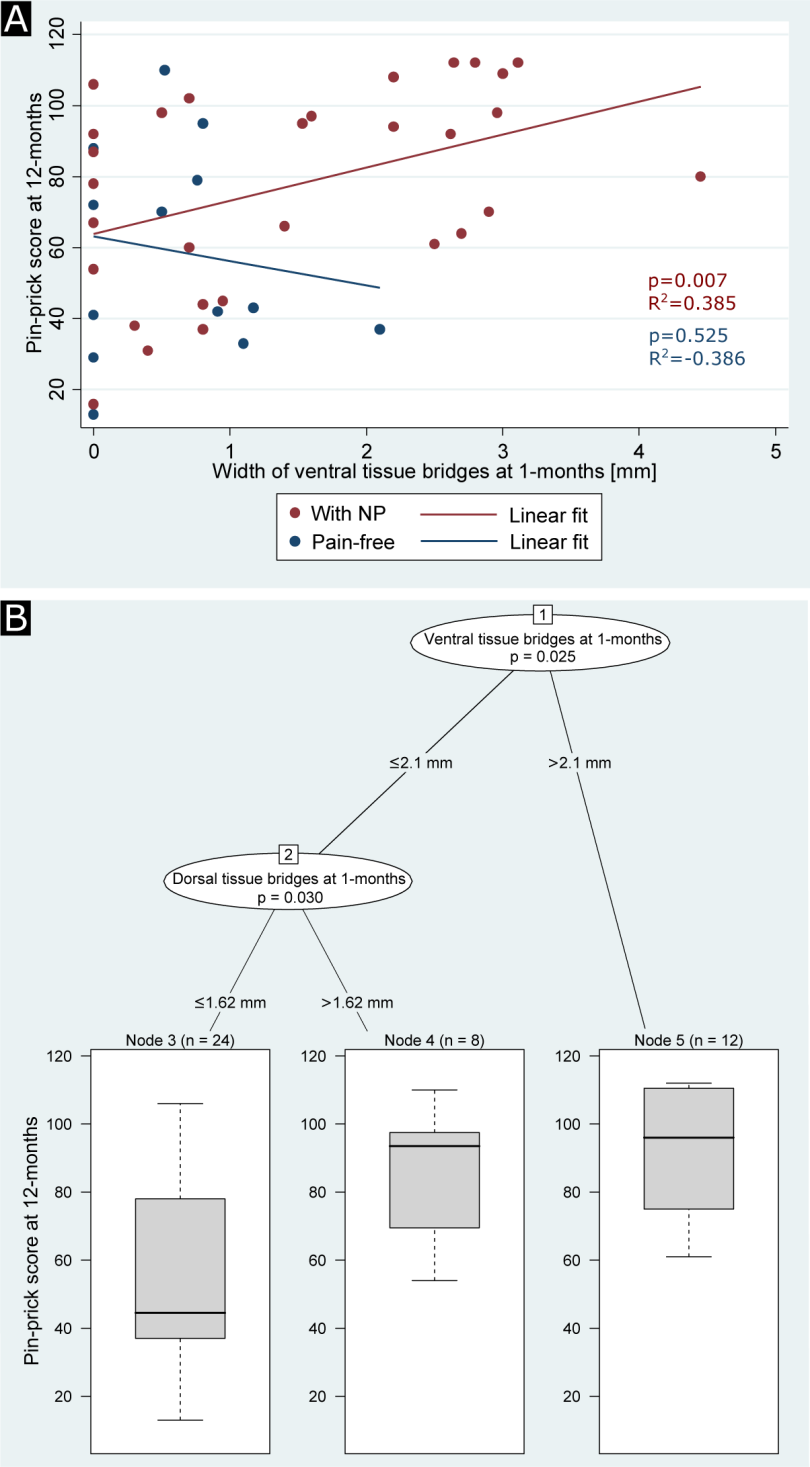
Regression analyses between width of tissue bridges at 1 month and pin-prick score at 12 months. (A) Regression graph showing the association of pin-prick score at 12 months with width of ventral tissue bridges at 1 month for patients with spinal cord injury with neuropathic pain (NP) (indicated in red) and pain-free patients (indicated in blue). The red and blue lines indicate the corresponding linear fits. (B) Unbiased recursive partitioning conditional inference tree for the clinical end point pin-prick score at 12 months of all patients with spinal cord injury (n=44). Ventral and dorsal tissue bridges’ widths at 1 month were used as predictors in the model. The algorithm led to a partition of the initial patient population (node 1) into one more inner node (node 2) and finally into three terminal nodes (nodes 3, 4 and 5) representing the more homogeneous subgroups. Box plots at the bottom show sizes of the subgroups, indicated above each terminal node, with their corresponding distributions (including two-sided error bars) of the clinical end point. Uncorrected p values are reported in (A) and multiple testing-corrected (ie, Bonferroni-corrected) p values are reported in (B) for significant differences.

Larger ventral tissue bridges at 1 month were associated with a higher mean NP intensity (coefficient=0.20, 95% CI 0.03 to 0.36, p=0.022, R^2^=0.131, n=42) and higher pin-prick score at 12 months (coefficient=0.03, 95% CI 0.01 to 0.04, p<0.001, R^2^=0.355, n=44), independent of dorsal tissue bridges, age and 1 month clinical score. When looking at subgroups separately, patients with NP (coefficient=0.03, 95% CI 0.01 to 0.04, p=0.007, R^2^=0.385, n=30, figure 4A), but not pain-free patients (coefficient=−0.01, 95% CI −0.03 to 0.02, p=0.525, R^2^=−0.386, n=14) showed a positive relationship between width of ventral tissue bridges at 1 month and pin-prick score at 12 months.

URP-CTREE analysis separated the entire cohort (p=0.042, n=42) into two terminal nodes with regard to the patients’ mean pain intensities at 12 months, according to the 1 month width of ventral tissue bridges being ≤2.1 mm (n=30, node 2) or >2.1 mm (n=12, node 3). The two subgroups presented 12 months mean pain intensities of 1.90±2.26 and 3.83±1.19, respectively. The URP-CTREE algorithm also identified 1 month ventral tissue bridges as a predictor for 12 months pin-prick score, its width separating the initial group (p=0.025, n=44, figure 4B) into two nodes with ≤2.1 mm (n=32, node 2) and >2.1 mm (n=12, node 5) in ventral tissue bridges’ width. The two subgroups presented 12 months pin-prick scores of 63.84±28.26 and 92.67±19.43, respectively. In a second step, the algorithm identified width of dorsal tissue bridges as a second predictor variable (p=0.030) for the subgroup showing a width of ventral tissue bridges ≤2.1 mm and separated it into two more nodes with ≤1.62 mm (n=24, node 3) and >1.62 mm (n=8, node 4) in dorsal tissue bridges’ width. The two subgroups presented 12 months pin-prick scores of 56.58±27.18 and 85.63±19.66, respectively.

## Discussion

This study demonstrates the potential involvement of spared ventral tissue bridges in the development and maintenance of NP following SCI. In particular, patients with NP had larger total and ventral tissue bridges when compared with pain-free patients, their width at 1 month being a predictor for a higher NP intensity and higher pin-prick score at 12 months post-SCI.

As expected,^2 31^ about two-third (68.2%) of the patients with SCI enrolled in this study developed NP of which more than half had already NP at the subacute stage (56.8%). Hari *et al*^16^ reported that enhanced recovery of spinothalamic tract function (ie, pin-prick) is associated with NP after SCI, and Hatem *et al*^32^ showed that there were less spinothalamic tract impairments in patients with syringomyelia with both spontaneous and evoked pain when compared with patients with spontaneous pain only. In our study, recovery of pin-prick score (ie, clinical measure for pain sensation) paralleled the emergence of NP and its intensity while no substantial change was observed in the pain-free group. This supports the notion that recovery of spinothalamic function is involved in the emergence and/or maintenance of NP.^8 16 32–34^

Ventral tissue bridge measures are likely to cover the anterior spinothalamic tract.^21^ Interestingly, patients with NP had greater width of ventral tissue bridges at baseline and follow-up, while the width of dorsal tissue bridges was similar between groups at both time points. Crucially, larger ventral tissue bridges at 1 month are associated with higher mean pain intensities at 1 month and 12 months, independent of dorsal tissue bridges. By means of URP-CTREE analysis, we further identified ventral tissue bridges at 1 month as predictors of recovery of pin-prick scores at 12 months with a cut-off value of 2.1 mm. Next to the implication of ventral tissue bridges, dorsal tissue bridges might also be involved in the generation of NP.^18 19^ To examine pin-prick sensation (ie, clinical pain measure), the patient needs to distinguish a sharp and a dull end of a pin when blindfolded.^22^ Thus, next to spinothalamic tract function, the medial lemiscal system running through the dorsal columns (ie, dorsal tissue bridges) is also involved as it conveys the information for tactile discrimination.^35^ Thus, dorsal tissue bridges likely involve second-order neurons of the spinothalamic tract within the substantia gelatinosa of the spinal cord dorsal horn.^36^ Sparing of these neurons could be of high importance for maintenance or recovery of clinical pain sensation (ie, pin-prick score) at dermatomes corresponding to the lesioned spinal cord levels. Finnerup *et al*^37^ reported that patients with SCI with NP, when compared with pain-free patients with SCI, showed more sensory hypersensitivity in lesion level-related dermatomes, suggesting a key role of sensory neuronal hyperexcitability in NP following SCI.^38–40^

This study has limitations. First of all, this study is a retrospective monocentric study with specified inclusion criteria. This likely represents a bias source as the study design resulted in a homogeneous data set, but might not reflect the general SCI population. Future studies would therefore benefit from a prospective multicentric design including larger patient numbers. Furthermore, women and men were not equally distributed in our patient cohort. However, this is also not the case for the general SCI population.^41^ Quantification of tissue bridges was only performed on midsagittal but not parasagittal slices. Further quantification of lateral tissue bridges on T2w axial slices was also not possible due to a low spatial resolution. This limits information about specific spared tracts and potential correlations with their corresponding functions. These points should be addressed in future studies to further explore the already promising method of tissue bridges characterisation^9–11 17^ with its reliable quantification already at early stages after SCI and even in the presence of metal artefacts near the lesion.^10^ The EMSCI pain questionnaire rather crudely assesses NP characteristics. Future studies would benefit from using more detailed pain assessments including drawings of the body with the extent, quality and intensity of the perceived pain at different body parts.^42^ Finally, even though our sample size was rather large, including more patients in the URP-CTREE analysis would increase the chances of reliably predicting future clinical end points and stratifying a heterogeneous patient cohort into homogeneous subgroups via multilevel inference trees and corresponding predictor variables.

This study identifies spared tissue bridges as quantifiable neuroimaging biomarkers assessed by conventional MRI predicting the emergence and maintenance of NP. Specifically, we identified 1 month width of ventral tissue bridges as the strongest predictor of NP intensity and pin-prick score at 12 months post-SCI. Thus, ventral tissue bridges represent MRI metrics, which could be readily implemented in the daily clinical routine to serve as promising, reliable and time-saving neuroimaging biomarkers for the monitoring of NP and for stratification of patient subgroups in interventional clinical trials.
